# New Cure for Hydrocele

**Published:** 1841-07

**Authors:** 


### A NEW CURE FOR HYDROCELE.

Colonel W. M., a gentleman of Smith County, Tenn., eminent for his public and private worth, aged about 75 years, had been the subject of hydrocele during nearly a third part of that time, and although the collection of the fluid had been slow, the tumor had attained a very uncomfortable size before he was relieved of it by an operation which, in this connexion, may be termed novel. In attempting to mount his horse, early in May last, his saddle turned and he fell on the other side upon his shoulders, by which his body received a smart concussion but no injury. He rose without difficulty, and proceeded to the place of religious worship where he was going, feeling only some slight pain and a sense of tension in the tumor along the course of the spermatic cord. A few nights after the fall he thought that he found less trouble than usual in disposing of the tumor preparatory to sleeping, but it did not occur to him that this was owing to its being diminished in size, nor was his attention called to this circumstance until a week after the accident, when on retiring to bed he was surprised at finding that it had entirely disappeared. In one week the collection of fluid, amounting, it is supposed, to a quart, was taken up by absorption, in consequence, it would seem, of a new action in the parts set up by the shock given by the fall. It has now been upwards of five weeks since the Colonel was thus most unexpectedly relieved of his infirmity, and there is yet no appearance of its return.

A circumstance followed the termination of this case which the physicians who attended the Colonel believed had some connexion with the absorption of the fluid. He was attacked, seven days after the tumor vanished, with profuse diarrhœa, his discharges resembling the rice-water passages in cholera. He was greatly prostrated by the disease, one of the most distressing symptoms of which was an uncontrollable propensity to sleep. He recovered gradually, and now enjoys excellent health. He is a man of strict temperance and regular habits, and is not conscious of having brought on the attack of diarrhœa by any indiscretion. His case is a curious one as to the mode in which he was relieved of the hydropic effusion, and not less so if it be admitted, that the discharge of the absorbed fluid from the system through the bowels was the cause of the diarrheœa, which, we confess, we are not quite prepared to do. Y.

